# Engineering Complexity in Bacterial Regulatory Circuits for Biotechnological Applications

**DOI:** 10.1128/mSystems.00151-17

**Published:** 2018-04-10

**Authors:** Cauã Antunes Westmann, María-Eugenia Guazzaroni, Rafael Silva-Rocha

**Affiliations:** a FMRP-University of São Paulo, Ribeirão Prêto, SP, Brazil; b FFCLRP-University of São Paulo, Ribeirão Prêto, SP, Brazil

**Keywords:** combinatorial gene regulation, CRISPR/Cas9, circuit engineering, regulatory elements, regulatory network, synthetic biology

## Abstract

Engineering microbial systems allows the generation of new technologies having significant impact in the biotechnological industry and on human health. In the past few years, several synthetic biology approaches have been implemented in bacteria to allow precise engineering of novel regulatory circuits for several applications. The advent of high-throughput technologies and clustered regularly interspaced short palindromic repeat (CRISPR)/Cas9-based DNA editing techniques have been pivotal in this process. Yet, despite the tremendous advances experienced recently, there are still a number of bottlenecks that need to be overcome in order to generate high-performance redesigned living machines, and the use of novel computer-aided approaches would be essential for this task. In this perspective, we discuss some of the main advances in the field of microbial engineering and the new technologies and approaches that should allow the construction of on demand synthetic microbial factories through the redesign of regulatory complexity.

## Abstract (precis)

Engineering microbial systems allows the generation of new technologies having significant impact in the biotechnological industry and on human health. In the past few years, several synthetic biology approaches have been implemented in bacteria to allow precise engineering of novel regulatory circuits for several applications.

## PERSPECTIVE

Microorganisms play a central role in human life, not only because they cause many diseases but also because they are used in several industrial processes. In addition to their critical importance for humankind, their fast growth in laboratory conditions and relatively easy genetic manipulation have allowed us to gather a tremendous amount of information regarding the molecular details of cell physiology and about the way microorganisms process information. In particular, the collection of this information in well-curated data banks and the advent of genomic and postgenomic approaches permitted the field of synthetic biology to emerge, initially based on microbial engineering (1). In the main workflow of synthetic biology, living cells are treated analogous to electronic devices, and cell engineering is executed through design-build-test cycles (1). In this sense, synthetic biology approaches are directed to modify the regulatory network of the cell, enabling organisms to perform novel molecular functions, with applications ranging from biosensing toxic or dangerous compounds, to searching for and killing pathogens and cancer cells, or producing compounds of interest, among many others (2). In this context, it is critical that the gene regulatory system of the target organism is modified to perform precise and reliable computations based on a set of inputs of interest, and for this, the analogy to logic gates is very useful (3, 4). Over the years, a considerable amount of effort has been dedicated toward the construction of reliable logic circuits, particularly in bacteria. For this, features such as noise in gene expression (5), composability of the biological parts used (6), signal detection range, and specificity of the circuits have been of special interest (2, 7). Thus, significant advancement has been experienced in the field, and along with it, our basic understanding of the molecular mechanisms related to the control of gene expression through the many cellular regulatory networks of interest has increased dramatically.

Most of the progress experienced in the field has been possible due to both technological and methodological improvements achieved in recent years. Of note are a number of high-throughput technologies (ranging from deep DNA sequencing techniques, microfluidics, automatization of molecular biology platforms, new DNA synthesis procedures, etc.) which have allowed not only large-scale analyses of genes and genomes but also the fast and precise, simultaneous construction and testing of several variants of synthetic circuits in living cells (6, 8). On the other hand, the recent rise of novel DNA editing techniques, with the indubitable central role of the clustered regularly interspaced short palindromic repeat (CRISPR)/Cas9-based approach, have allowed the enhanced modification of native organisms, either to add minor changes in its regulatory networks or to introduce entirely new synthetic circuits (9). In addition to DNA editing, the CRISPR/Cas9 technologies have permitted editing-independent activation or repression of genes based on the usage of Cas9 mutant variants not able to perform DNA cleavage. In these methods, the guide RNA (gRNA) is designed to target the promoter of the gene of interest, allowing either the recruitment of the RNA polymerase (RNAP) for activation of the target promoter or the blockage of binding of the RNAP, leading to gene repression (10). This type of approach is revolutionary, since it allows easy targeting of the gene of interest without the necessity of laborious engineering of the binding specificities of transcriptional factors (TFs) by amino acid mutagenesis.

Despite the remarkable improvement undergone in the field of synthetic biology, a number of bottlenecks are emerging. First, most of the engineered circuits are limited to one or few inputs, and usually the construction of more-complex circuits requires several TFs and cognate promoters, leading to final circuits composed of several kilobases of DNA fragments. For example, a recently engineered synthetic circuit allowing Escherichia coli to sense and respond to three wavelengths of light (red, green, and blue) required the engineering of around 48 kb of rewired genetic material (11). This feature strongly limits the complexity of the circuit that can be engineered and implemented in the host cell. Second, most of the synthetic biology work has focused on model organisms such as E. coli and Saccharomyces cerevisiae for which there are well-established genetic tools. Yet, many applications relevant to biotechnology or with biomedical significance require a different type of host (or chassis), more adapted to the final environment of interest. For instance, bacteria engineered to invade cancer cells focus on the usage of Salmonella enterica serotype Typhimurium (12), while a more metabolically robust bacterium such as Pseudomonas putida would be required for industrial applications with process-specific parameters (13). Finally, the number of biological parts well characterized for synthetic biology applications is very limited, and more variants have to be mined from different sources.

Regarding the first bottleneck, one potential solution would be the generation of novel technologies allowing the compression of as many regulatory interactions in the shortest DNA fragment as possible. In this “logic compression” approach, many different TFs would recognize the same promoter, and the result of their interaction would define the expression level and expression dynamics of the gene of interest. It is worth mentioning that this approach is completely different from the synthetic circuits based on cascade of TFs. In fact, some recent progress has been made in this direction. By using genetic algorithms based on the recognition of patterns of well-known TFs, it has been possible to engineer synthetic promoters simultaneously recognized by two or three TFs, demonstrating the potential of computer models to generate functional, new (new to nature), regulatory elements in bacteria (14, 15). While these initial works have focused on overlapping binding sites for different TFs, the construction of promoters with arrays of binding sites would potentially generate very complex regulatory patterns. In order to understand the regulatory rules necessary for the construction of these complex promoters, we recently characterized several synthetic complex promoters in E. coli. By using this approach, we unexpectedly found that complex promoters containing arrays of binding sites for different TFs are prone to emergent properties, in which the combination of simple TF-binding sites generates an expression behavior that cannot be predicted on the basis of the individual behaviors (16). This finding has strong implications not only for the engineering of complexity in bacteria but also for our understanding in how gene regulation operates in response to multiple signals in natural systems (17).

As discussed before, the main limitation in the use of alternative hosts for synthetic biology is the lack of suitable genetic tools for genetic manipulation of these organisms. In this context, there has been tremendous progress in the development of novel genetic tools for the engineering of nonmodel and biotechnologically relevant organisms such as the cyanobacterium *Synechococcus* sp. strain PCC 7002 (18), the Gram-positive bacterium Streptomyces venezuelae (19), the yeast Pichia pastoris (20), as well as many phylogenetically unrelated Gram-negative bacteria (21). The list of organisms for which new-generation genetic tools are available is growing at an impressive speed every year, and use of these new tools would allow the construction of even more complex circuits in several organisms of interest. Finally, as the number of well-characterized biological parts is very limited, a potential solution could be searching for new parts in microbial genomes or metagenomes, thus allowing the expansion of the portfolio of applications that could be performed in the field (22). Attempts to overcome this bottleneck include the use of synthetic circuits to identify new functional biological parts, such as small-metabolite transporters (23) or regulatory sequences capable of inducing gene expression in response to compounds of interest (24–26). These types of approaches are quite interesting, since synthetic circuits can be used to mine functional elements that in turn can be used to construct novel engineered strains.

In summary, we propose that major efforts in the field should be directed to the development of novel approaches focused toward engineering of complex regulatory logic in shorter DNA fragments, either by assembling arrays of CRISPR/Cas9 regulatory modules or by constructing TF-based complex promoters. Consequently, this approach should reduce the limitations of handling large DNA constructions and shortcomings due to the propagation of expression noise in cascades of TFs. Additionally, increasing the diversity of microbial chassis using novel genetic tools will be essential for the implementation of the final circuits into hosts more suitable for the final application of interest. Finally, the further development of novel theoretical concepts imported from electronic engineering, such as control theory and retroactivity (27, 28), should enhance the design of synthetic circuits with reliable performance and robust dynamics. These characteristics are crucial to allow the construction of novel microbial systems having significant impact in biotechnology.
